# Exploring the Distinct Effect of Age at Onset and Caudate Denervation on Cognitive Deficits in Early Parkinson’s Disease

**DOI:** 10.14336/AD.2024.0553

**Published:** 2025-06-11

**Authors:** Giovanni Palermo, Sara Giannoni, Luca Tommasini, Gabriele Bellini, Daniela Frosini, Gayane’ Aghakhanyan, Riccardo Morganti, Duccio Volterrani, Nicola Pavese, Roberto Ceravolo

**Affiliations:** ^1^Center for Neurodegenerative diseases - Parkinson’s disease and Movement disorders, Unit of Neurology, Department of Clinical and Experimental Medicine, University of Pisa, Pisa, Italy.; ^2^Regional Center of Nuclear Medicine, Department of Clinical and Experimental Medicine, University of Pisa, Pisa, Italy.; ^3^Section of Statistics, University of Pisa, Pisa, Italy.; ^4^Clinical Ageing Research Unit, Translational and Clinical Research Institute, Newcastle University, Newcastle upon Tyne, United Kingdom.; ^5^Department of Clinical Medicine, Nuclear Medicine and PET, Aarhus University, Aarhus, Denmark.

**Keywords:** age, caudate, cognitive deficits, dopamine transporter, Parkinson’s disease

## Abstract

Older age at onset and baseline caudate dopaminergic denervation are recognized risk factors for cognitive impairment in Parkinson’s disease (PD), posing challenges in identifying their relative contribution to cognitive outcomes. The objective of this study was to assess the distinct contribution of age at onset and baseline caudate dopaminergic binding to the early cognitive deficits in PD patients. We examined the relationship between baseline dopaminergic striatal dysfunction (measured using [^123^I]-FP-CIT SPECT), age at disease onset and neuropsychological performance in 128 *drug-naive* PD patients, utilizing putaminal and caudate binding values of 77 healthy controls (HC) for a comparative exploration of age-dependent loss of DAT availability. Additionally, we investigated whether age at onset and DAT binding value of the caudate could independently predict cognitive changes over a median of 7-year follow-up. [^123^I]-FP-CIT-SPECT binding values had a significant negative correlation with age in both PD and HC, but in PD, aging was linked with a steeper slope for the caudate than the putamen. Older age at onset and lower caudate uptake were associated with worse global cognitive function and performance in specific neuropsychological tests at baseline and demonstrated to be significant independent predictors of cognitive dysfunction at follow-up. Our findings confirm a differential age effect on [^123^I]-FP-CIT binding in the striatal subregions of *de novo* PD patients. Notably, we found less age-related attrition of dopaminergic binding in the putamen than in the caudate, reflecting likely the superimposition of putaminal compensatory mechanisms and an increased predisposition of old onset PD patients to develop cognitive disturbances.

## INTRODUCTION

Parkinson's disease (PD) is a common neurodegenerative disorder clinically characterized by motor and non-motor symptoms [1]. Cognitive impairment is a debilitating and frequent comorbidity of late-stage PD, with a cumulative incidence approaching 80% in some community studies [2]. However, even newly diagnosed PD patients may exhibit cognitive deficits [3,4], which are indicative of worse overall symptoms and serve as a strong predictor for the development of dementia [5]. While motor deficits in PD are associated with the extensive loss of dopaminergic neurons in the substantia nigra pars compacta (SNpc), the underlying pathophysiology of early cognitive impairment remains incompletely understood [6]. There is consensus that dopamine-mediated striato-frontal circuits are critically implicated in multiple cognitive functions, mainly executive abilities [7] and that caudate dysfunction contributes to cognitive deficits since the early stages of PD [8]. Nevertheless, the dopaminergic dysfunction in PD follows a typical posterior-to-anterior gradient with a relative sparing of the head of the caudate in the early stages of the disease [9]. Older age at PD onset represents another significant risk factor for early cognitive involvement [10]. Cognitive skills and dopaminergic neurotransmission also decline in the process of aging [11-13]. Interestingly, the influence of age on presynaptic dopamine transporter (DAT) levels in PD appears more pronounced in the caudate than in the putamen, suggesting a disease-independent effect of age-related caudate dysfunction in early cognitive deficits [14,15].

Based on these premises, rather than revisiting the largely established role of advanced age at PD onset and caudate dopaminergic denervation in PD-related cognitive deficits, our objective was to evaluate the potential association between early cognitive decline in these patients and the decline in caudate DAT availability that is associated with the normal aging process. In this regard, we examined a large cohort of *de novo* PD patients, incorporating extensive neuropsychological data, with the aim to explore the relationship between caudate dopaminergic dysfunction at baseline, age at PD onset and cognitive deficits. Additionally, our goal was to comprehend the distinct contribution of age at onset and baseline caudate binding to the development of longitudinal early cognitive deficits also describing the incidence of mild cognitive impairment (MCI) and dementia in a cohort of PD patients during the 7 years’ clinical follow-up. A better understanding of the relationship between age of onset and striatal dopaminergic denervation at baseline might be helpful to gain further insights into the underlying mechanisms of cognitive deficits in PD patients.

## MATERIALS AND METHODS

### Study participants

A total of 128 drug-*naive* PD subjects (56 women and 72 men, all of Caucasian ethnicity, with a mean age at inclusion of 65.45±7.8 years) were consecutively selected from patients referred to the Movement Disorders Clinic at Santa Chiara Hospital, Pisa University, between 2011 and 2013. PD diagnosis followed the UK Parkinson’s Disease Society Brain Bank [16] confirmed by [^123^I]-ioflupane-fluoropropyl-carbomethoxy-3-beta-4-iodo-phenyl tropane SPECT ([^123^I]FP-CIT-SPECT). None of the patients were on antidepressants or antipsychotic medications at our first evaluation (baseline). All subjects had a brain magnetic resonance imaging to exclude significant atrophy, white matter lesions, and lacunes in basal ganglia. To focus on idiopathic PD, atypical signs, PD gene mutations, other neurologic diseases, and the use of antidepressant or antipsychotic therapies were additional exclusion criteria. Patients were also recruited if they had been assessed at least once annually since their initial diagnosis and had a minimum follow-up period of three years. Standard neurological evaluations, including Hoehn and Yahr (H&Y) scale, the Unified Parkinson’s Disease Rating Scale (UPDRS), the Hamilton Depression and Anxiety Rating Scale (HAM-D and HAM-A) and the Mini Mental State Examination (MMSE) were conducted at each visit. Levodopa equivalent dose (LED) was calculated according to Tomlinson and colleagues [17] and the recent proposed conversion for safinamide and opicapone [18]. Baseline evaluation also included a detailed neuropsychological battery, as described below, in addition to the MMSE and FAB to ensure a comprehensive assessment of cognitive function. Importantly, both our baseline evaluation (comprehensive of neurological and cognitive assessment) and DAT-SPECT had to be performed within 24 months of patients' recollection of first symptoms (age at symptom onset).

For the retrospective longitudinal study, annual MMSE (or Montreal Cognitive Assessment -MoCA- scores when available) were collected from the medical records of the treating neurologists. A second neuropsychological battery was repeated during the follow-up period in those patients who developed clinically significant cognitive impairment, based on all available information (clinical interview of the patients and informant, and evidence of cognitive decline as screened by MMSE/MoCA scales). Diagnoses of mild cognitive impairment (PD-MCI) and dementia (PDD) were made according to published criteria [19,20].

The research was done in accordance with the Helsinki declaration and the local ethics committee, and the patients provided informed consent for the collection of data.

### Neuropsychological assessment

The neuropsychological evaluation consisted of MMSE and Frontal Assessment Battery (FAB) as screening instruments for overall cognition and executive function, respectively. A battery of standardized neuro-psychological tests was used. It consisted of digit span forward and Corsi block-tapping task to evaluate verbal and spatial short-term memory respectively; Rey Auditory Verbal Learning Test (RAVLT) and Rey-Osterrieth Complex Figure Test (ROCFT) for learning and long-term memory; copy of ROCFT to assess spatial organization, planning and visuo-constructional functions; Trail Making Test part A, part B and B-A for processing speed and set-shifting; Stroop Color/Word Test for inhibitory control; Raven’s Coloured Progressive Matrices (CPM), Modified Wisconsin Card Sorting Test -categories achieved and perseverative error- (mWCST- CA/PE) and Letter Fluency Test to explore abstract reasoning and cognitive flexibility. Raw neuropsychological scores were adjusted for age and education according to the normative data available for each cognitive test. A cut-off score of less than two standard deviations below normative data determined impaired performance on a neuropsychological test.

### DAT-SPECT protocol

[^123^I]FP-CIT-SPECT (DaTSCAN®, GE Healthcare, UK) was injected intravenously at a dose of approximately 185 MBq, according to standard procedure. Scans were acquired between 3 and 4 hours after tracer injection. Subjects were scanned with a dual-head gamma camera (Discovery 710; GE Healthcare, Milwaukee, WI, USA) equipped with high resolution, low energy parallel hole collimators. Acquisition parameters were as follows: circular orbit > 360 degrees, 120 projections (angular sampling: 3 degrees), matrix 128 × 128, pixel size 3 mm, and overall scanning time of 35 to 40 minutes. SPECT reconstruction was performed using the ordered subset expectation maximization (OSEM) algorithm (2 iterations, 10 subsets) and by applying a three-dimensional (3D) post-reconstruction filter (Butterworth; order 10, cut-off, 0.5 cycles/cm) and attenuation correction (Chang method, μ = 0.12 cm^-1^).

[^123^I]FP-CIT binding ratios were calculated using the BasGan V2 software, which allows for precise, semi-quantitative analysis of DaT SPECT images. BasGan V2 enables automatic 3D segmentation of the caudate nucleus and putamen using a high-definition, 3D striatal template derived from Talairach’s atlas, and it generates a 3D occipital region of interest (ROI) for background evaluation. The software also incorporates partial volume effect correction in the binding computation process. The BasGan V2 software and its methods are detailed in Zubal et al. [21] and the software's user manual (BasGan V2 User Manual, 2010) provides further reference.

Caudate nucleus and putamen [^123^I]FP-CIT binding was subtracted by background as follows: Striatal binding ratio: [(caudate nucleus or putamen [^123^I]FP-CIT uptake -background uptake)/background uptake] to obtain specific to non-displaceable binding ratios (SBR) of the caudate nucleus and the putamen of each hemisphere. In each patient, caudate nucleus and putamen with the largest reduction of [^123^I]FP-CIT binding were referred as the most affected caudate and most affected putamen whereas the contralateral striatal nuclei were defined as the least affected caudate and putamen.

For validation of the binding ratios and a comparative exploration of age-dependent loss of DAT binding in subjects without PD, [^123^I]FP-CIT-SPECT binding values were obtained from 77 healthy controls (HC) included in the European database of healthy controls (ENC-DAT). The ENC-DAT database is a well-established reference that has been validated in multiple studies, providing a reliable benchmark for our measurements [22]. The control group was meticulously selected to match the gender and age distribution of our study population, ensuring minimal confounding effects of these factors on dopaminergic binding. To assess the lateralization of SBR changes, as additional parameter, striatal asymmetry index (AI) of the caudate and putamen SBR was calculated using the following formula [(AI)=([(A-B)/(A+B)] x 2 x 100) where A corresponds to the SBR within the ROI of the striatum (caudate or putamen) contralateral to the most severely affected side, and B to the SBR within the ROI of the striatum ipsilateral to the most severe side of the body [23]. Then, we also calculated the caudate/putamen ratio (C/P ratio) as: SBR CAUDATE MOST/SBR PUTAMEN IPSI, where MOST refers to the caudate with the lowest SBR value and IPSI to the putamen of the same hemisphere [24].

### Statistical analysis

Statistical analysis was performed using the SPSS software program (version 29 for MS windows. Chicago, USA). Continuous variables were presented as means and standard deviations, and nominal data as numbers and percentages (Table 1). Student's t-test assessed differences between PD and HC in age, [^123^I]FP-CIT binding values and neuropsychological scores among PD-MCI patients and those without cognitive deficits.

Simple linear regression examined the relationship between striatum binding ratios (SBRputamen and SBRcaudate) and age at onset. The slopes in each subregion were compared between PD and healthy controls using a multiple linear regression model with interaction terms. Simple linear regression also assessed the correlation between SPECT variables (caudate and putamen uptake individually) or age at PD onset with neuropsychological scores. Multiple regression analysis followed, with neuropsychological scores as the dependent variable and age at PD onset and caudate SBR binding values as independent variables.

The impact of common demographic and clinical variables at baseline on the risk of MCI/dementia during the follow-up was investigated using both univariate and multivariate Cox regression analysis (patients with MCI at baseline were not included). Any variables showing significant associations in univariate analysis with p-values less than 0.05 were included in the multivariate model. Statistical significance was set at p=0.05.

**Table 1:** Demographic and clinical features of PD patients included at baseline.

Numbers	128
Age at symptom onset (yrs)	64.4±8.1
Age at baseline (yrs)	65.45±7.80
Female/Male n. (%)	56/72 (43.8%-56.2%)
Education (years)	10.29±4.36
Duration of disease at diagnosis (months)	12.08±9.96
UPDRS-II	5.47±3.54
UPDRS-III	17.95±7.94
Motor phenotype at baseline	
Tremor-dominant	41 (32.0%)
Akinetic-rigid	53 (41.4%)
Mixed	34 (26.5%)
Predominant side of motor symptoms	
Right	46 (35.9%)
Left	50 (39.1%)
Bilateral	32 (25.0%)
PD-MCI n. (%)	28 (21.8%)

Data are expressed as mean ± standard deviation (for continuous variables) or absolute and relative frequency (for categorical variables). Abbreviations: MCI, mild cognitive impairment; UPDRS, Unified Parkinson's disease rating scale.

## RESULTS

### Characteristics of patients at baseline

The Table 1 presents detailed demographic and clinical characteristics of PD patients. Most of patients were males (56.2%). The average onset age was 65.4 years (SD, 7.8 years), and the median follow-up period was 7 years (SD, 2.2 years). Age at DAT-SPECT in the HC group did not significantly differ from patients (Table 2).

Clinical assessment and neuropsychological tests (Table 3) revealed that at baseline, 28 patients (21.9%) had MCI, while none had dementia. The PD-MCI subgroup, compared to patients without early cognitive disturbances, was older at their first visit (69.3±6.9 vs 64±7.7; p=0.002) and showed significantly lower [^123^I]FP-CIT binding values in the caudate nucleus (3.25±0.89 vs 3.75±0.84, p=0.02) (Table 4). Although DAT availability was also lower in the putamen of early MCI patients compared to cognitively unimpaired subjects, this trend did not reach statistical significance (1.67±0.46 vs 1.93±0.56, p=0.057) (Table 4).

**Table 2:** [^123^I]FP-CIT binding values of PD patients and healthy controls.

	Patients	HC (n. 77)	p value
Age at baseline (yrs)	65.45±7.80	64.07±9.56	0.24
Female/Male n. (%)	56/72 (43.8%-56.2%)	32/45 (41.5%-58.4%)	0.75
Caudate SBR			
most affected	3.35±0.86	-	-
least affected	3.88±0.93	-	-
mean	3.62±0.87	5.27±0.97	<0.0001
Putamen SBR			
most affected	1.62±0.58	-	-
least affected	2.08±0.65	-	-
mean	1.87±0.54	4.75±0.88	<0.0001
C/P ratio	2.00±0.35	1.15±0.32	<0.0001
Caudate AI (%)	14.78±9.83	-	-
Putaminal AI (%)	26.50±19.46	-	-

Data are expressed as mean ± standard deviation (for continuous variables) or absolute and relative frequency (for categorical variables). Abbreviations: AI, asymmetry index; C/P, Caudate/putamen; HC, healthy controls; SBR, Specific Binding Ratio.

**Table 3:** Baseline neuropsychological assessment of PD patients.

Cognitive Tests	Score	Norms cutoff score
FAB	15.44±1.88	<13.5
MMSE	27.60±2.04	<23.8
Digit Span Forward	5.59±0.76	<3.75
Corsi's block-Tapping Test	4.69±0.72	<3.5
RAVLT Immediate Recall	37.67±7.45	<28.53
RAVLT Delayed Recall	7.75±2.31	<4.69
ROCFT Copy	31.42±4.87	<23.76
ROCFT Immediate Recall	15.91±6.35	<6.44
ROCFT Delayed Recall	15.18±5.94	<6.33
Letter Fluency Test	34.31±9.10	<17.35
CPM	29.46±4.78	<18.96
Stroop Color/Word Test IE-T	17.80±13.23	>36.91
Stroop Color/Word Test IE-E	0.42±1.53	>4.23
Trail Making Test A	54.41±30.17	>93
Trail Making Test B	86.40±55.64	>282
Trail Making Test B-A	35.35±47.75	>186
mWCST-CA	5.09±1.33	<3
mWCST-PE	2.49±2.99	>6.40
**Behavioral Measures**		
HAM-D	4.27±4.78	>13
HAM-A	4.09±5.03	>10

Data are expressed as mean ± SD. Abbreviations: CPM, Raven’s Coloured Progressive Matrices; FAB, Frontal AssessmentBattery; HAM-A, Hamilton Anxiety Rating Scale; HAM-D, Hamilton Depression Rating Scale; IE-T, Interference Effect-Time; IE-E, Interference Effect-Error; MMSE, Mini Mental State Examination; mWCST- CA/PE, Modified Wisconsin Card Sorting Test -Categories Achieved/Perseverative Error; RAVLT, Rey Auditory Verbal Learning Test; ROCFT: Rey-Osterrieth Complex Figure Test; SD: standard deviation

### Striatal binding of [^123^I]FP-CIT in PD patients and healthy controls

**Figure 1.**
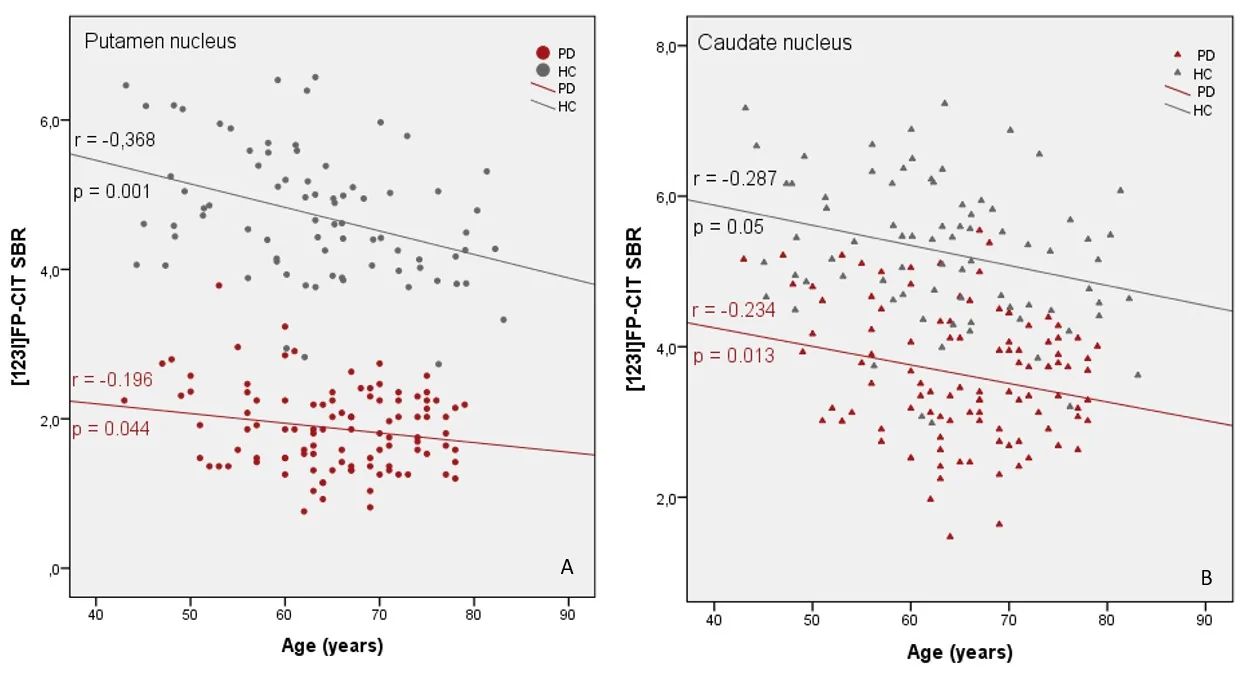
Effects of age: comparison of [^123^I]FP-CIT binding values between PD and HC in the putamen (A) and caudate (B).

As expected, [^123^I]FP-CIT SBR was significantly lower in patients compared to HC (p<0.0001 for both caudate and putamen) (Table 2). Similarly, when expressed as the C/P ratio (an index of the posterior-to-anterior dopaminergic loss gradient), higher values were found in PD patients than in HC, indicating more severe dopamine depletion in the putamen than in the caudate nucleus in early PD (Table 2). Striatal DAT binding values showed a significant negative correlation with age in both PD and healthy subjects (Fig. 1).

**Figure 2.**
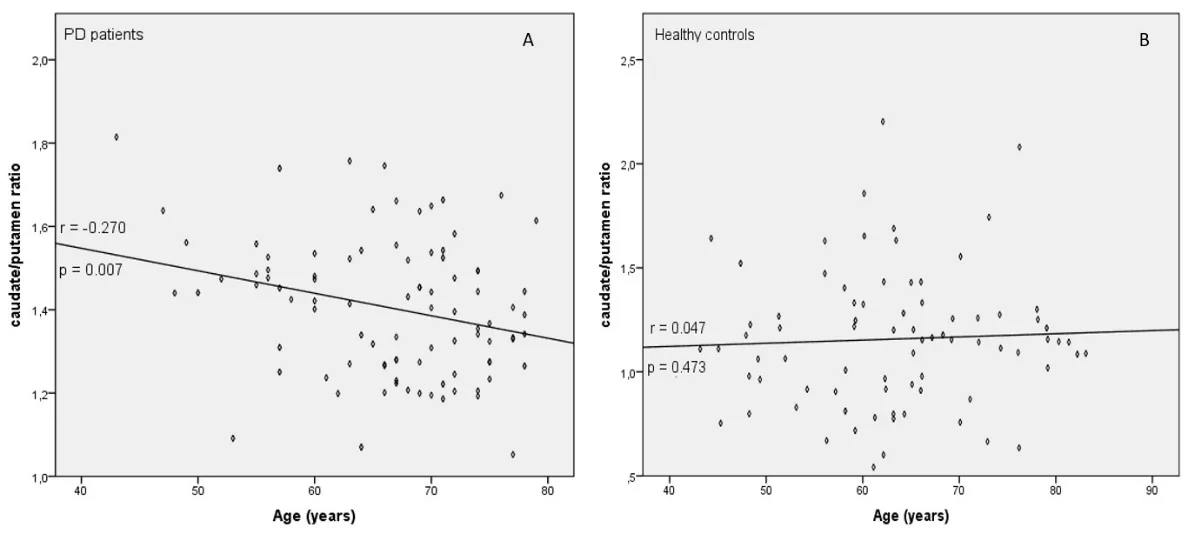
Effects of age on C/P (Caudate/putamen) ratio in PD (A) and HC (B).

In HC, there was a significant linear effect of age on striatal uptake, decreasing by -3.3% per decade in the caudate (B=-0.022; p=0.05) and -4.9% per decade in the putamen (B=-0.034; p=0.001). Similarly, SBR showed a significant inverse correlation with age in the PD group, declining in both the caudate and putamen. Older PD patients tended to have lower mean striatal [^123^I]FP-CIT binding values than younger subjects in both caudate and putamen, but in PD, aging was associated with a steeper slope for the caudate (-4.0% per decade in the caudate vs 3.7% per decade in the putamen) (Fig. 1). Consistent with this, the slopes of regression were different between PD and healthy control groups in the putamen(p=0.09), showing a higher age-related decline in HC than in PD patients, but not in the caudate(p=0.886). Accordingly, the C/P ratio showed age-related shrinkage only in PD patients, while there was no age effect on the C/P ratio in HC (Fig. 2).

**Table 4:** Differences between age, [^123^I]FP-CIT binding values and neuropsychological scores between patients with MCI and without cognitive disturbances at baseline.

	PD-MCI (n. 28)	PD with normal cognition (n. 100)	p value
**Age at baseline (yrs)**	69.32±6.966	64.05±7.73	0.002*
**UPDRS-III**	20.48±7.96	17.56±7.84	0.099
**Uptake values**			
Mean Caudate SBR	3.25±0.89	3.75 ±0.84	0.020*
Mean Putamen SBR	1.67±0.46	1.93±0.56	0.057
**Neuropsychological tests**			
FAB	13.41±2.40	15.96±1.31	0.0001*
MMSE	25.89±2.09	27.99±1.76	0.0001*
Digit span Forward	5.38±0.65	5.65±0.75	0.110
Corsi's block-Tapping Test	4.80±0.75	4.61±0.69	0.217
RAVLT Immediate Recall	28.14±7.40	40.04±7.25	0.001*
RAVLT Delayed Recall	6.51±1.77	8.15±2.40	0.001*
ROCFT Copy	22.92±7.80	33.27±2.23	0.001*
ROCFT Immediate Recall	12.74±7.65	16.97±5.77	0.003*
ROCFT Delayed Recall	10.51±6.53	16.68±5.14	0.0001*
Letter Fluency Test	31.03±11.49	35.00±8.19	0.098
CPM	26.33±5.82	30.24±4.14	0.003*
Stroop Color/Word Test IE-T	25.09±18.31	16.23±10.77	0.021*
Stroop Color/Word Test IE-E	1.21±2.75	0.20±0.83	0.065
Trail Making Test A	76.15±41.94	47.38±21.59	0.002*
Trail Making Test B	131.73±76.88	73.13±42.68	0.002*
Trail Making Test B-A	66.82±73.48	26.56±36.08	0.017*
mWCST-CA	4.19±1.53	5.35±1.12	0.003*
mWCST-PE	4.09±3.26	2.08±2.77	0.007*

Data are expressed as mean ± standard deviation.Abbreviations: Abbreviations: CPM, Raven’s Coloured Progressive Matrices; FAB, Frontal Assessment Battery; IE-T, Interference Effect-Time; IE-E, Interference Effect-Error; MMSE, Mini Mental State Examination; mWCST- CA/PE, Modified Wisconsin Card Sorting Test -Categories Achieved/Perseverative Error; RAVLT, Rey Auditory Verbal Learning Test; ROCFT: Rey-Osterrieth Complex Figure Test; SBR, Specific Binding ratio; UPDRS: Unified Parkinson’s Disease Rating Scale.*p<0.05

**Figure 3.**
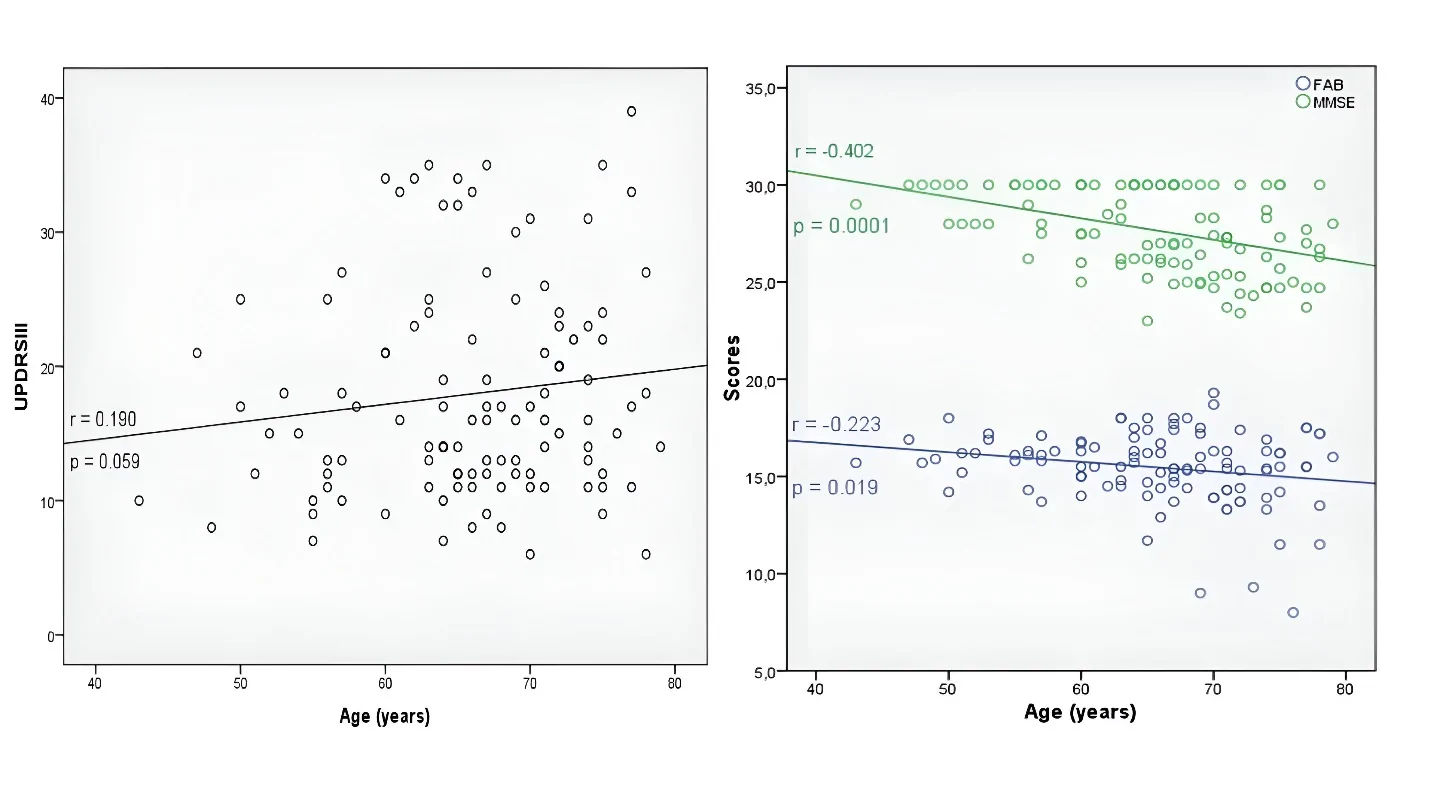
Scatterplot showing association between age at PD onset and UPDRS III score (on the left), and between age at PD onset and measures of general cognitive function (Mini-Mental State Examination -MMSE- and Frontal Assessment Battery -FAB-) (on the right).

### Relationship between age at PD onset and [^123^I]FP-CIT binding values with clinical features at baseline

In the PD group, older age at onset correlated with increased motor impairment, although not statistically significant (p=0.059, Fig. 3), and reduced global cognitive function measured by the MMSE and FAB scales (p=0.0001 and p=0.019, respectively, Fig. 3). Additionally, a significant age-related decline was observed in the Digit Span Forward (p=0.019), copy of ROCFT (p=0.007), Trail Making Test part B (p=0.05), and mWCST CA/PE (p=0.004/p=0.043) in the PD population (Table 5).

**Table 5:** Correlation between age at PD onset and neuropsychological scores at baseline (adjusted for age and level education).

Neuropsychological Tests	Age	
	r	p value
FAB	-0.223*	0.019*
MMSE	-0.402*	0.0001*
Digit Span Forward	-0.216*	0.019*
Corsi's block-Tapping Test	0.016	0.856
RAVLT Immediate Recall	-0.080	0.372
RAVLT Delayed Recall	-0.030	0.741
ROCFT Copy	-0.245*	0.007*
ROCFT Immediate Recall	-0.023	0.796
ROCFT Delayed Recall	-0.021	0.818
Letter Fluency Test	-0.041	0.646
CPM	-0.090	0.326
Stroop Word/Color Test IE-T	-0.039	0.664
Stroop Word/Color Test IE-E	0.125	0.163
Trail Making Test A	0.154	0.092
Trail Making Test B	0.181*	0.050*
Trail Making Test B-A	0.166	0.073
mWCST-CA	-0.296*	0.004*
mWCST-PE	0.208*	0.043*

Abbreviations: CPM, Raven’s Coloured Progressive Matrices; FAB, Frontal Assessment Battery; IE-T, Interference Effect-Time; IE-E, Interference Effect-Error; MMSE, Mini Mental State Examination; mWCST- CA/PE, Modified Wisconsin Card Sorting Test -Categories Achieved/Perseverative Error; RAVLT, Rey Auditory Verbal Learning Test; ROCFT: Rey-Osterrieth Complex Figure Test. *p<0.05

**Figure 4.**
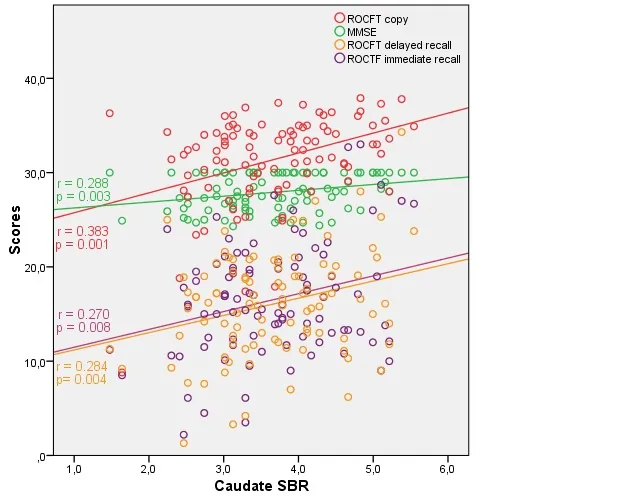
Scatterplot showing association between baseline [^123^I]FP-CIT SPECT binding values in the caudate and cognitive scores.

Age-adjusted caudate [^123^I]FP-CIT binding values were positively correlated with MMSE (p=0.003) and ROCFT (copy, p<0.001; Immediate Recall, p=0.008; Delayed Recall, p=0.004) (Fig. 4 and Table 6). Conversely, putaminal baseline DAT uptake showed no significant correlation with cognitive tests (Table 7). However, age-adjusted SBR values of the putamen exhibited an inverse significant correlation with the UPDRS III (r=0.266; B=-3.82; p=0.027). In the multivariate regression model, both lower DAT density in the caudate and older age at onset remained significant independent predictors of poor performance on the MMSE (p=0.03 and p=0.0001, respectively) and on specific neuropsychological tests (Digit Span Forward and mWCST-CA for age; ROCFT Copy, Immediate and Delayed Recall for caudate SBR) (Table 8).

### Follow-Up Evaluation

In the follow-up study, 19 patients were excluded from the analysis due to short or irregular follow-up periods, as it was deemed necessary to ensure the reliability and accuracy of the longitudinal analysis. This resulted in a final cohort of 109 patients for the longitudinal assessment. During the follow-up period, 26 new cases of MCI emerged in the PD cohort (20.3% of the overall PD sample), while 18.8% (24/109) progressed to clinically diagnosed dementia. Of those with PD-MCI, 42.9% converted to dementia. Multivariate Cox regression confirmed older age at onset and lower caudate [^123^I]FP-CIT SBR as independent significant predictors of MCI (p=0.009 and p=0.022) and dementia (p=0.026 and 0.014) (Table 9).

**Table 6:** Correlation between neuropsychological scores (adjusted for age and level education) and[^123^I]FP-CIT binding values in the caudate nucleus.

Neuropsychological Tests	Caudate SBR	
	r	p value
FAB	0.121	0.259
MMSE	0.288*	0.003*
Digit Span Forward	0.019	0.856
Corsi's block-Tapping Test	-0.083	0.409
RAVLT Immediate Recall	0.088	0.379
RAVLT Delayed Recall	0.015	0.882
ROCFT Copy	0.383*	0.001*
ROCFT Immediate Recall	0.270*	0.008*
ROCFT Delayed Recall	0.284*	0.004*
Letter Fluency Test	-0.002	0.988
CPM	0.052	0.612
Stroop Word/Color Test IE-T	0.023	0.821
Stroop Word/Color Test IE-E	-0.113	0.257
Trail Making Test A	-0.027	0.791
Trail Making Test B	0.077	0.461
Trail Making Test B-A	0.074	0.477
mWCST-CA	0.170	0.145
mWCST-PE	-0.092	0.435

Abbreviations: CPM, Raven’s Coloured Progressive Matrices; FAB, Frontal Assessment Battery; IE-T, Interference Effect-Time; IE-E, Interference Effect-Error; MMSE, Mini Mental State Examination; mWCST- CA/PE, Modified Wisconsin Card Sorting Test -Categories Achieved/Perseverative Error; RAVLT, Rey Auditory Verbal Learning Test; ROCFT: Rey-Osterrieth Complex Figure Test; SBR, Specific Binding ratio. *p<0.05

## DISCUSSION

This study aimed to examine the distinct contribution of baseline caudate DAT availability and age at PD onset to early cognitive deficits in a large cohort of *de novo* patient cohort followed up for a median of 7 years. Untangling the interaction between older age at onset and caudate dopaminergic denervation as reported risk factors for cognitive impairment in PD can be challenging. Aging is associated with changes in dopaminergic neuro-transmission, including the loss of pre- and postsynaptic biochemical markers [25,26]. There have been a few studies specifically investigating the effects of aging on DAT imaging in PD and reporting variable results [13]. Our findings are consistent with the dopaminergic cell loss observed in pathological studies [27] as well as with the percentages of DAT binding decline reported in other SPECT studies [13,28,29]. Unlike healthy aging, we observed a smaller decrease in DAT binding values with age in the putamen than in the caudate in PD patients.

**Table 7:** Correlation between neuropsychological scores (adjusted for age and level education) and putaminal [^123^I]FP-CIT uptake.

Neuropsychological Tests	Putamen SBR	
	r	p value
FAB	0.150	0.160
MMSE	0.186	0.092
Digit Span Forward	0.091	0.388
Corsi's block-Tapping Test	0.065	0.517
RAVLT Immediate Recall	0.014	0.890
RAVLT Delayed Recall	0.147	0.141
ROCFT Copy	0.120	0.257
ROCFT Immediate Recall	0.109	0.280
ROCFT Delayed Recall	0.155	0.124
Letter Fluency Test	0.080	0.424
CPM	0.018	0.861
Stroop Word/Color Test IE-T	0.063	0.532
Stroop Word/Color Test IE-E	0.126	0.209
Trail Making Test A	0.043	0.673
Trail Making Test B	0.031	0.767
Trail Making Test B-A	0.033	0.752
mWCST-CA	0.071	0.544
mWCST-PE	0.056	0.635

Abbreviations: CPM, Raven’s Coloured Progressive Matrices; FAB, Frontal Assessment Battery; IE-T, Interference Effect-Time; IE-E, Interference Effect-Error; MMSE, Mini Mental State Examination; mWCST- CA/PE, Modified Wisconsin Card Sorting Test -Categories Achieved/Perseverative Error; RAVLT, Rey Auditory Verbal Learning Test; ROCFT: Rey-Osterrieth Complex Figure Test; SBR, Specific Binding ratio. *p<0.05

In a [^18^F]FP-CIT PET study involving 39 *drug-naïve* PD patients, a similar aging effect in the striatum was observed, with a slower decline in the putamen compared to the caudate when compared with age-related changes in healthy control subjects [14]. This regional difference may indicate that PD involves different patterns of dopamine loss in the brain compared to normal aging. Indeed, both the caudate and putamen seem to be similarly affected in healthy aging [27]. Additionally, it may also suggest the activation of compensatory mechanisms in the nigrostriatal pathway in PD which could vary across different subregions of the basal ganglia with age. It is well-known that in PD adaptive mechanisms, such as DAT downregulation, come into play to partially compensate for the loss of dopaminergic terminals and delay the clinical onset of motor signs in the early stage of the disease [30-32]. The core motor features of PD largely reflect the degree of dopamine depletion in the putamen which is the most affected region in early PD evolution [33]. Consequently, compensatory mechanisms are expected to be most pronounced in the putamen, where fiber loss is profound also in the presymptomatic phase [34], while they are likely negligible in the caudate during early PD where dopaminergic denervation becomes apparent later [35].

**Table 8:** Multiple linear regression models examining the effects of age at PD onset and mean caudate SBR at baseline on neuropsychological test results (dependent variables).

Age at PD onset			Mean caudate SBR		
Neuropsychological tests	B	p value	B	p value	Adjusted R^2^
FAB	-0.184	0.096	0.071	0.519	0.024
MMSE	-0.380*	0.0001*	0.201*	0.03*	0.204
Digit Span Forward	-0.241*	0.026*	-0.046	0.668	0.033
Corsi's block-Tapping Test	-0.023	0.827	-0.088	0.396	-0.013
RAVLT Immediate Recall	-0.027	0.793	0.081	0.434	-0.012
RAVLT Delayed Recall	0.001	0.995	0.015	0.885	-0.020
ROCFT Copy	-0.144	0.141	0.347*	0.001*	0.149
ROCFT Immediate Recall	0.065	0.519	0.287*	0.005*	0.058
ROCFT Delayed Recall	0.097	0.337	0.309*	0.003*	0.071
Letter Fluency Test	-0.001	0.989	-0.002	0.985	0.020
CPM	-0.077	0.469	0.031	0.769	-0.013
Stroop Word/Color Test IE-T	0.045	0.663	0.034	0.743	-0.018
Stroop Word/Color Test IE-E	0.073	0.482	-0.095	0.358	-0.002
Trail Making Test A	0.142	0.181	0.007	0.949	-0.001
Trail Making Test B	0.201	0.058	0.123	0.242	0.023
Trail Making Test B-A	0.184	0.083	0.117	0.270	0.017
mWCST-CA	-0.247*	0.038*	0.105	0.374	0.060
mWCST-PE	0.183	0.130	-0.043	0.720	0.013

Abbreviations: B stand: standardized B Coefficient; CPM, Raven’s Coloured Progressive Matrices; FAB, Frontal Assessment Battery; IE-T, Interference Effect-Time; IE-E, Interference Effect-Error; MMSE, Mini Mental State Examination; mWCST- CA/PE, Modified Wisconsin Card Sorting Test -Categories Achieved/Perseverative Error; RAVLT, Rey Auditory Verbal Learning Test; ROCFT: Rey-Osterrieth Complex Figure Test; SBR, Specific Binding ratio. *p<0.05

Evidence suggests that older PD subjects exhibit less efficient compensatory mechanisms, including increased DAT levels, compared to those with earlier age of onset [15]. Similarly, a notable absence of compensatory changes in dopaminergic activity has been progressively documented in normal aging [36]. Hence, more effective putaminal presynaptic compensatory mechanisms in younger patients may contribute to the observed gradual slope of age-related changes in the putamen compared to the caudate in PD. The reduced aging effect in putaminal DAT binding could also be attributed to a more severe pathological loss of DA transporters in early onset PD [37], consistent with a probable floor effect in the putamen observed in PD patients. However, this would contradict the finding of lower UPDRS-III scores in patients with earlier age at onset. This is noteworthy as the UPDRS motor score is a reliable predictor of neuronal loss in the SNpc [38] and putaminal dysfunction remains closely related to motor dysfunction even in our PD cohort.

**Table 9:** Associations of baseline variables with MCI/dementia as determined by Cox proportional hazards models.

Event: MCI		Univariate Analysis				Multivariate Analysis				
*Baseline Variables*	B	HR	95% CI - lower	95% CI - upper	p-value	B	HR	95% CI - lower	95% CI - upper	p-value
Mean Caudate SBR	-0.871	0.418	0.223	0.784	**0.007**	-0.790	0.454	0.231	-0.892	0.022
Age	0.089	1.093	1.029	1.161	**0.004**	0.085	1.089	1.021	1.161	**0.009**
Sex [(0) F, (1) M]	0.727	2.070	0.882	4.856	0.095					
Education	-0.185	0.831	0.740	1.155	0.102					
Disease duration	-0.041	0.960	0.897	1.028	0.239					
Motor Phenotype	-0.444	0.641	0.345	1.194	0.161					
UPDRS-III	0.035	1.035	0.986	1.087	0.160					
HAM-D	0.027	1.027	0.911	1.157	0.664					
HAM-A	-0.036	0.965	0.837	1.112	0.620					
**Event: Dementia**		**Univariate Analysis**				**Multivariate Analysis**				
*Baseline variables*	B	HR	95% CI - lower	95% CI - upper	p-value	B	HR	95% CI - lower	95% CI - upper	p-value
Mean Caudate SBR	-0.793	0.452	0.253	0.808	**0.007**	-0.547	0.579	0.311	0.941	**0.014**
Age	0.074	1.077	1.009	1.150	**0.026**	0.094	1.098	1.012	1.193	**0.026**
Sex [(0) F, (1) M]	0.237	1.267	0.563	2.854	0.568					
Education	-0.165	0.848	0.758	1.146	0.118					
Disease duration	-0.003	0.997	0.953	1.043	0.902					
Motor Phenotype	-0.224	0.799	0.449	1.422	0.445					
UPDRS-III	0.066	1.068	1.015	1.123	0.011	0.056	1.058	0.994	1.126	0.079
HAM D	0.040	1.040	0.924	1.172	0.514					
HAM A	-0.040	0.961	0.824	1.121	0.612					

Abbreviations: HAM-D/HAM-A, Hamilton Depression and Anxiety Rating Scale; HR, Hazard Ratio; MCI, Mild Cognitive Impairment; SBR, Specific Binding Ratio; UPDRS, Unified Parkinson’s Disease Rating Scale *p<0.05

On the other hand, the distinct trajectory of DAT binding in the caudate across the age range of onset reported in our PD patients could imply a specific age-related effect on the dopaminergic function of the caudate nucleus, different from normal aging and independent of disease duration. The more pronounced decline in caudate dopamine concentration with age in PD could contribute to the predisposition to cognitive impairment in patients with late-onset disease. Furthermore, early caudate dopaminergic dysfunction is not uncommon among patients with PD at the onset of parkinsonian symptoms, and other studies suggest that individuals exhibiting reduced tracer binding in both caudate nuclei are older than the control group [8].

Normal aging encompasses various cognitive changes including decline in episodic memory, executive functions, spatial ability, divided attention and speech production [39]. A loss of gray matter in several cortical and subcortical brain structures, including the striatum, is observed in aging individuals [40]. Notably, the caudate undergoes significant age-related volumetric loss along with cognitive decline in older adults [41,42]. Structural alterations could be accompanied by molecular changes as demonstrated by the pronounced impact of age on dopaminergic presynaptic markers in the caudate over the putamen [43] and decreased synaptic density limited to the caudate nucleus (1.7%/decade) [44]. Thus, the caudate appears to lie at the intersection of the correlative triad among aging, dopamine, and cognition [45]. It has prominent non-motor connections with higher-level cognitive areas (e.g. dorsolateral prefrontal cortex, rostral anterior cingulate, and inferior frontal gyri) and plays a primary role in planning and executing strategies and behaviors required for achieving complex goals [46]. Such “executive deficits'' are specific to PD patients, reflecting dopaminergic dysfunction in fronto-striatal networks caused by dopamine depletion in the dorsal caudate nucleus [47,48], but also closely resemble those observed in aging [49].

In our cohort, older age at onset and baseline reduced caudate DAT uptake were independently associated with early generalized cognitive dysfunction and poorer performance in specific neuropsychological tests (ROCFT copy, Trail Making Test part B and mWCST and Digit Span Forward) mainly related to the integrity of the frontal executive domain. Similarly, 21.9% of our subjects with MCI at the time of our first evaluation had both significantly reduced DAT availability in the caudate and an older age at onset compared to patients with no cognitive deficits in the baseline neuropsychological battery. Furthermore, in the longitudinal analysis, older age at onset and lower caudate DAT binding were both predictors of MCI and dementia. Although unraveling the complex relationship between DAT availability and age at PD onset poses challenges, age may partially mediate the association between DAT availability in the caudate and early cognitive decline, contributing to the more pronounced dopaminergic denervation observed in older individuals with PD. Whether the impact of the caudate is direct or indirectly influenced by age, our findings reinforce the key role of the caudate in initiating cognitive deficits in the early stages of PD [50]. Several SPECT studies using dopaminergic tracers have confirmed the role of nigro-caudate dysfunction in cognitive impairment associated with PD, even in *drug-naïve* patients [51-54]. However, in these studies, we cannot entirely dismiss the possibility that age independently and significantly influenced both the cognitive status of patients with PD and DAT binding, even though age was controlled for in some of them. Yet, a rigorous study by Seipel and colleagues [55] found that age moderated the effect of DAT binding on executive deficit, and the mediation effect was stronger for younger subjects than for older. Although it would have been interesting to conduct a similar analysis in our study and concurrently investigate the association between caudate uptake and the neuropsychological performance of HC, the latter was not available in our controls. However, the present study aimed not to explore the well-known association of caudate binding and age at onset with cognitive decline in PD or to investigate the pathophysiological basis of cognitive deficits in PD, but rather to determine whether age has a different effect on striatal dopaminergic function between controls and PD patients, disentangling the differential contribution of age at onset and dopaminergic caudate denervation in the occurrence of early cognitive disturbances. Our results confirm a greater striatal dopaminergic dysfunction on DaTSCAN in older PD patients compared to early onset PD [37], with a more pronounced age-related loss in the caudate vs. putamen compared to HC [14,56]. We may speculate that this difference may be attributed to the age-related loss of compensatory DAT downregulation in the putamen, which parallels the superimposed age effect observed in the caudate.

Hence, it is plausible that a combination of PD-specific and aging-related dopamine depletion in the caudate could contribute to the risk and degree of early cognitive dysfunction, according to the hypothesis that *“the superposition of the effects of the disease process and aging may involve an interaction at the level of the underlying causes or mechanisms, such that the combined effect of the 2 processes in terms of neuronal loss in relevant structures is greater than the sum of the separate effects*” [57].

Among the strengths of our study, we must mention the inclusion of a large sample of de novo patients within 2 years from symptom onset, and with a comprehensive neuropsychological assessment at baseline. Importantly, this retrospective but longitudinal study also provides insights into the DAT-dependent long-term prognosis of cognitive performance. Nonetheless, the lack of more detailed segmentation of the striatum represents a limitation. This study is subject to other constraints as well. First, it is a single-center study, and we acknowledge the need for multicenter studies to enhance generalizability. Secondly, the involvement of non-dopaminergic neurotransmitter systems and other factors, such as Alzheimer disease-like pathology and cerebrovascular disease, should be acknowledged as potentially relevant to understanding the biological basis for age-related cognitive deficits in PD. Although patients with monogenic parkinsonism were not included, the genetic background itself may act as an additional confounding factor in the current results. It is plausible that the association between cognitive impairment and reduced caudate DAT uptake in our study might reflect the overall decline in dopaminergic function with increasing disease severity. However, it is noteworthy that disease duration was similar at both baseline and follow-up for patients with and without MCI. Lastly, differences in dopaminergic treatment could have influenced longitudinal changes in cognitive function, although the LED at the last follow-up did not differ between cognitively stable and worsened subjects (not shown). Furthermore, we acknowledge that lifestyle factors (such as physical activity, diet, and smoking), medication adherence, and comorbid conditions, could also act as potential confounding factors affecting cognitive outcomes. Future studies should aim to systematically control these variables to better isolate the effects of dopaminergic binding and aging on cognitive function in PD.

Our results validate the association between older age at PD onset and caudate denervation with specific aspects of cognitive impairment in PD, elevating the likelihood of both early and late cognitive complications. Importantly, our study enhances the understanding of the intricate interplay between PD-specific and aging-related dopamine depletion in the caudate, supporting a superimposed age effect at onset. This likely contributes to the heightened susceptibility of older PD patients to early cognitive disturbances.

Future research should also consider larger, multicenter cohorts to enhance the generalizability of findings and the systematic control of a broader range of potential confounding factors, including lifestyle and genetic factors. This will help in better understanding the intricate relationship between dopaminergic function and cognitive outcomes in PD.
